# Evaluating the Safety of Herbal Medicine on Renal Function: A Comprehensive Analysis from Six Randomized Controlled Trials Conducted with Four Formulations from Traditional Korean Medicine

**DOI:** 10.3390/ph17050544

**Published:** 2024-04-23

**Authors:** Jin-Yong Joung, Chang-Gue Son

**Affiliations:** 1 Department of Internal Medicine, Daejeon Good-Morning Oriental Hospital, Dunsan-ro 123 beon-gil 21, Seo-gu, Daejeon 35240, Republic of Korea; bluetxt1@gmail.com; 2 Department of Korean Medicine, Korean Medical College, Daejeon University, Daehak-ro 62, Dong-gu, Daejeon 34520, Republic of Korea; 3 Liver and Immunology Research Center, Daejeon Oriental Hospital of Daejeon University, Daedukdae-ro 176 bun-gil 75, Seo-gu, Daejeon 35235, Republic of Korea

**Keywords:** herb-induced renal injury, drug-induced renal injury, adverse drug reaction, nephrotoxicity

## Abstract

The growing popularity of herbal medicine raises concerns about potential nephrotoxicity risks, while limited evidence hinders a comprehensive impact assessment. This study aims to investigate the overall risk features of herbal medicine on kidney injury. We conducted a retrospective analysis on renal function changes, including blood urea nitrogen (BUN), serum creatinine, and estimated glomerular filtration rate (eGFR), through data from six randomized controlled trials (RCTs) in South Korea. A total of 407 participants (142 males, 265 females) received either one of four different herbal medicines (240 participants) or a placebo (167 participants). When comparing changes in eGFR regarding the mean, 90th-percentile value, and 20% reduction after treatment, there was no significant difference between the herbal-treated and placebo groups. This study provided a helpful reference for examining the safety issues of herbal remedies, especially regarding kidney function.

## 1. Introduction

The integration of herbal medicine into daily diets, through functional foods and supplements, has been widely adopted worldwide, reflecting a growing interest in natural remedies and their potential health benefits. However, alongside their therapeutic potentials, concerns regarding the safety of these herbal compounds, particularly in terms of hepatotoxicity and nephrotoxicity, have emerged [1,2]. Recently, numerous studies have focused on the safety level of herbal medicine for liver function [3,4,5]. Meanwhile, the incidence rate of herb-induced liver injury (HILI) has been estimated to be around 0.6% among 1001 inpatients in 10 Korean-medicine hospitals [6]. However, current knowledge regarding nephrotoxicity related to herbal medicine is limited due to a lack of scientific evidence.

Drug-induced kidney injury (DIKI) is a major adverse drug reaction, accounting for a significant proportion of acute kidney injury cases worldwide, with an incidence of 1%–5% [7]. Herbal drug-induced kidney injury (HIKI) depends on various factors, such as the specific herb, the dose, the duration of use, and an individual’s overall health [8]. Certain herbs, for instance, those containing aristolochic acid, are known to pose risks for renal failure and urinary tract cancer [9]. *Echinacea purpurea* and *Hypericum perforatum* have been used in the treatment of upper respiratory infections and depressive disorder, respectively, due to their immunomodulatory properties and ability to regulate neurotransmitters [10,11]. However, they also could induce kidney dysfunction when taken in high doses or for extended periods [12,13].

Kidney dysfunction impacts over 10% of the global population, affecting more than 800 million people [14]. Its prevalence is rising due to factors like chronic disease prevalence, aging populations, and environmental influences. Kidney dysfunction is recognized by the World Health Organization (WHO) as a significant global health issue, with increasing mortality and disease burden [15]. Treatment options are limited, and side effects from conventional methods, such as dependency on dialysis and long-term usage of angiotensin-converting-enzyme (ACE) inhibitors or diuretics, may lead to complications like electrolyte imbalances and blood pressure variations, affecting patients’ quality of life [16]. Herbal medicine presents a potential alternative or complementary therapy, especially as several herbs have shown kidney-protective and function-improving effects, suggesting new avenues for kidney dysfunction treatment strategies [17,18,19].

Meanwhile, practitioners of conventional medicine frequently call for stringent regulations on traditional herbal treatments, highlighting concerns over risks not limited to specific herbs but stemming from improperly managed toxicity and the presence of heavy metals across a wide range of traditional remedies [20]. Particularly in East Asian countries such as China, Korea, Taiwan, and Japan, where traditional herbal medicine is an integral part of the health insurance system, there is a growing emphasis on conducting risk assessment studies to evaluate these treatments more rigorously [21]. Despite this increased scrutiny, the comprehensive impact of traditional herbal medicine on renal function remains largely unexplored. 

South Korea features a dual healthcare system that seamlessly integrates Western and Traditional Korean Medicine (TKM). Licensed practitioners can prescribe herbal medicines within TKM, which are also covered by national health insurance. This integration ensures that such treatments are not only widely utilized but also culturally ingrained within the healthcare system [22]. Additionally, herbal supplements like red ginseng are readily available at various retail outlets, including pharmacies. Moreover, traditional herb markets and health food stores provide crude herbs, which are commonly used by individuals to create non-professional, homemade decoctions.

This study aims to expand our knowledge to assess the overall risk of herbal drugs for kidney injury using data from six randomized controlled trials (RCTs) previously conducted in South Korea by our research group. These trials included four formulations from TKM. We performed a retrospective analysis focusing on essential renal function indicators: blood urea nitrogen (BUN), serum creatinine, and estimated glomerular filtration rate (eGFR). Our evaluation focused on the impact of these herbal medicines on kidney function, specifically analyzing changes in the mean and 90th-percentile eGFR values, along with notable instances of eGFR reduction. Despite its preliminary nature, this research represents the first concerted effort to systematically evaluate the safety of herbal medicines on kidney function. As such, it lays the groundwork for future investigations into HIKI, an area that is of growing concern but is currently limited by a dearth of scientific evidence.

## 2. Results

### 2.1. Participants Characteristics

A total of 407 participants (142 males and 265 females) were enrolled, distributed between 240 in the intervention group and 167 in the placebo group. The overall mean age of participants was 44.2 ± 11.1 years (ranging from 18 to 73 years), with the intervention group averaging at 44.0 ± 10.7 years and the placebo group at 44.5 ± 11.8 years, showing no significant difference between the two. The average BMI was 22.9 ± 3.0, with the intervention group at 22.7 ± 3.0 and the placebo group at 23.1 ± 2.9, also indicating no significant disparity. In the six RCTs, participants received one of four different herbal drugs: CGX for chronic liver disease (65 participants), BST for functional dyspepsia (97 participants), Myelophil for chronic fatigue syndrome (79 participants), and *Panax ginseng* extract for healthy (78 participants) or fatigued condition (88 participants). Details regarding participant characteristics, including the distribution of age, BMI, and number of individuals per treatment group, are shown in Table 1.

### 2.2. Changes in BUN, Serum Creatinine, and eGFR

**Figure 1:**
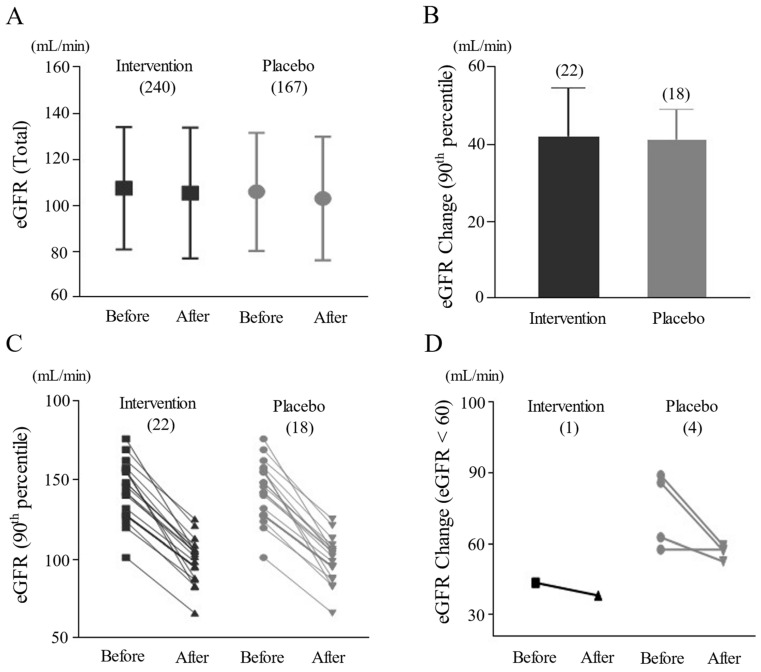
(**A**). Comparison of eGFR before and after treatment (mean ± SD). (**B**). Changed value in eGFR in the 90th-percentile analysis after treatment (mean ± SD). (**C**). Comparison of eGFR changes in the 90th-percentile analysis. (**D**). Comparison of eGFR changes in participants with eGFR < 60 after treatment. Number of participants indicated as (n). No significant changes in BUN, creatinine, and eGFR were noted post-treatment between two groups.

No notable difference in the total mean values between the two groups (intervention vs. placebo) was observed regarding BUN (from 13.1 ± 3.3 to 13.5 ± 7.0 vs. 13.5 ± 3 to 13.3 ± 3.4), serum creatinine (from 0.81 ± 0.17 to 0.84 ± 0.17 vs. 0.81 ± 0.17 to 0.83 ± 0.18), and eGFR (from 107.2 ± 26.7 to 105.2 ± 28.5 vs. 105.8 ± 25.5 to 102.8 ± 26.7), respectively (Figure 1A and Table 2).

Analysis by gender showed no significant changes between the two groups for BUN (male: from 13.9 ± 3.5 to 13.7 ± 4.2 vs. 14.3 ± 3.2 to 13.4 ± 3.0; female: from 12.6 ± 3.0 to 12.6 ± 3.4 vs. 13.1 ± 3.6 to 13.2 ± 3.6), serum creatinine (male: from 0.91 ± 0.16 to 0.91 ± 0.19 vs. 0.92 ± 0.18 to 0.94 ± 0.18; female: from 0.75 ± 0.14 to 0.78 ± 0.16 vs. 0.76 ± 0.14 to 0.79 ± 0.15), and eGFR (male: from 93.6 ± 19.7 to 95.9 ± 24.4 vs. 93.2 ± 20.5 to 91.8 ± 23.5; female: from 115.4 ± 27.1 to 110.8 ± 29.4 vs. 111.6 ± 25.5 to 108.0 ± 26.7). Additionally, analysis by treated herbal drugs indicated no significant changes between the groups across different treatments: CGX, BST, Myelophil, and ginseng. Further details are shown in Table 2. 

Among the 407 participants enrolled in the study, there were no reports of significant adverse reactions, including acute kidney failure, which is defined as an increase in serum creatinine of 0.3 mg/dL or more within 48 h, a serum creatinine increase of 1.5 times the baseline or previous week’s value, or urine output less than 0.5 mL/kg/h for 6 h [23].

### 2.3. Change in eGFR in 90th-Percentile and Frequency of Notable Reduction

When we analyzed the 90th-percentile of eGFR reduction after medication (40 of 407 participants, 22 from intervention and 18 from placebo group), the mean change was 41.9 ± 12.7 in the intervention versus 41.3 ± 7.7 in the placebo group, with no significant statistical difference (Figure 1B,C and Table 3). The number of participants with an eGFR less than 60 declined from four before to one after herbal medication. The remaining participant was a 69-year-old male whose eGFR dropped from 43.1 to 37.6 after taking BST for 4 weeks to treat functional dyspepsia. Meanwhile, the number of cases of eGFR less than 60 increased from three to four in the placebo group (Figure 1D and Table 3). In the herbal-drug group, 18.3% (44 of 240 participants) experienced a decrease of 20% or more in eGFR after medication, while in the placebo group, the rate was 21.0% (35 of 167 participants). Among these, the number of cases with eGFR dropping below 60 post-treatment was zero in the intervention group and two in the placebo group (Table 3).

## 3. Discussion

From the analysis of data derived from six RCTs on kidney function in South Korea, we estimated the risk of kidney injury associated with four distinct herbal medicines from TKM. Three multi-herbal drugs, Myelophil, BST, and CGX, showed partial benefits for patients experiencing symptoms of chronic fatigue syndrome (CFS) [24], functional dyspepsia [25,26], and liver fibrosis [27], respectively, in our RCTs. *Panax ginseng* extract alleviated mental fatigue and oxidative stress markers in two RCTs, one targeting healthy volunteers [28] and the other targeting chronic fatigue patients [29]. No significant adverse events were observed in these six RCTs, regarding both subjective complaints and laboratory findings.

In contrast to the well-established diagnostic criteria for DILI, such as the Roussel Uclaf Causality Assessment Method (RUCAM) score [30], there are no universally accepted criteria for DIKI. The eGFR is a key index of renal function that measures the kidney’s filtration capacity, and thus is usually adapted to judge DIKI [31]. Because there was no notable change in average eGFR level after drug administration compared with before, we utilized the 90th-percentile analysis, considering only the top 10% of participants exhibiting eGFR reductions. This 90th-percentile analysis is a statistical method often employed, when appropriate criteria are absent and the frequency rate of abnormality is rare, to determine any potential concerns [32,33]. Our data revealed no significant differences between the 90th-percentiles of both the intervention and placebo groups (41.9 ± 12.7 vs. 41.3 ± 7.7), indicating no harmful effects of our four herbal drugs, at least, on kidney function (Figure 1B,C and Table 3). This finding was supported by an additional analysis of cases in which eGFR decreased by 20% or more and fell below 60 (Figure 1D and Table 3). In general, an eGFR decrease of ≥20% after administration or an eGFR below 60 following drug administration is a threshold recognized as indicative of deteriorating kidney function [34]. These criteria had a compelling rationale to identify instances where any drug may lead to potential risks [35].

The prevalence of DIKI is notably higher in specific patient demographics, especially those with known risk factors such as dehydration, sepsis, renal dysfunction, cardiovascular disease, and diabetes. Infants and young children are also at a heightened risk [36]. Additionally, the use of certain medications, including nonsteroidal anti-inflammatory drugs (NSAIDs), antibiotics, and chemotherapy agents, is associated with an increased prevalence of DIKI [37]. The participants in this study were enrolled according to the criteria of their respective RCT, which led to the exclusion of a considerable number of individuals with risk factors for DIKI from the study population. Therefore, the findings of this study may underestimate the actual risk of HIKI in clinical practice. 

Females are known to exhibit a 1.5 to 1.7 times higher risk of experiencing adverse drug reactions compared to males, a trend that is also observed in the incidence of DILI [38]. Similarly, a nationwide prospective study of 1001 patients treated with herbal drugs found that all six cases of HILI occurred exclusively in women, further indicating a gender-specific vulnerability [6]. This increased vulnerability in females to DILI can be attributed to sex differences in pharmacokinetics, pharmacodynamics, hormonal influences, and immune responses. Despite the well-documented gender disparities in DILI and HILI, the area of DIKI still lacks comprehensive research on gender-based vulnerability. However, gender-specific susceptibilities have been observed with certain drugs; for example, cisplatin nephrotoxicity is more prevalent in perimenopausal women [39], whereas in animal models, cyclosporine A has been shown to cause more severe nephrotoxic effects in male rats [40]. Our study, limited by its small size and absence of severe renal injury cases, found no significant gender differences in kidney function decline, hindering definitive conclusions on gender’s influence on HIKI susceptibility.

The underlying mechanisms of DIKI are multifaceted, encompassing direct nephrotoxic effects from chemotherapy agents like cisplatin, alterations in hemodynamics caused by non-steroidal anti-inflammatory drugs, inflammatory responses triggered by antibiotics, and crystal deposition resulting from the use of drugs like acyclovir [31]. The roles of inflammation and oxidative stress in the progression of DIKI are highlighted by markers such as tumor necrosis factor-alpha (TNF-α), interleukin-1 beta (IL-1β), and interleukin-6 (IL-6) for inflammation and advanced glycation end-products (AGEs) and malondialdehyde (MDA) for oxidative stress, which have been documented to significantly contribute to DIKI [41,42]. However, our study did not observe these inflammatory and oxidative stress markers typically linked to nephrotoxicity, emphasizing the need for further research to investigate these indicators in the context of nephrotoxicity induced by herbal medicines.

Knowledge about the molecular mechanisms of herbal nephrotoxicity remains limited. Nonetheless, three primary factors are recognized for elevating the risk of HIKI: substantial blood flow through the kidneys, their intense metabolic activity, and the reabsorption of glomerular filtrate by renal tubules, leading to high intracellular agent concentrations [43]. Aristolochic acid, a compound found in plants belonging to the Aristolochiaceae family and recognized for its nephrotoxicity, has been found to cause kidney damage by inducing oxidative stress, apoptosis, and inflammation, ultimately leading to fibrosis [44]. The herbal medicines investigated in our study do not include the medicinal herbs known to be involved with nephrotoxicity. Recent studies have also highlighted the potential for interactions between herbal and conventional drugs, which could aggravate nephrotoxicity, emphasizing the need for further investigation in this area [9].

In contrast to concerns about the nephrotoxicity of certain herbal compounds, it is noteworthy that specific herbal medicines have been recognized for their potential to protect against kidney injury and enhance renal function [45,46]. For example, *Polyporus umbellatus* and *Poria cocos*, types of mushrooms frequently employed in TKM, exhibited protective and function-enhancing effects on the kidneys. These benefits are linked to their capabilities in modulating immune responses, providing anti-inflammatory advantages, and offering antioxidative protection [47,48]. However, it is important to note that our RCTs were not specifically designed to improve renal function, and individuals with pre-existing renal impairments were excluded, possibly explaining the lack of observed enhancements in renal function.

This present study has several limitations. Firstly, the herbal medicines used in this study were standardized formulae, supported by preliminary safety data from animal studies. However, in the real-world practice of TKM, many herbal prescriptions are decoctions with compositions and dosages tailored to individual needs and traditional practices, thus limiting the generalizability of our findings to practical clinical settings. Additionally, it is estimated that over 30% of herbal users in South Korea consume these substances without a professional TKM prescription, which could increase the risk of adverse effects [22]. Generally, herbal formulations prescribed by TKM practitioners are known to be safer due to stricter regulations, quality control, and appropriate use [49]. However, this study overlooked the fact that a significant portion of the population may be at risk due to non-professional prescriptions, and it did not account for the potential nephrotoxic effects of these improperly used herbal drugs.

Secondly, the research was conducted within a specific geographical context (South Korea) and by a single research team, which may constrain the extrapolation of our findings across different populations and ethnic groups. Thirdly, the retrospective nature of our analysis may limit our ability to establish causality between the use of herbal medicines and kidney health outcomes, highlighting the need for further prospective investigations. Lastly, the study’s focus on short-term herbal medicine’s effects and reliance on a small sample size presents limitations. This leaves the long-term persistence of these effects unclear and raises questions about the robustness and generalizability of our findings. Future inquiries should include larger sample sizes and longer study durations to understand these effects more comprehensively, underlining the necessity for ongoing research in this area.

## 4. Materials and Methods

### 4.1. Study Design and Ethics Approval

In this study, we conducted a retrospective analysis of data collected from six RCTs we previously conducted using 4 different herbal drugs for durations from 4 to 12 weeks. The primary objective was to assess the impact of herbal medicine on kidney function by comparing the changes in renal function-related values, including BUN, serum creatinine, and eGFR, between the intervention and placebo groups. BUN levels were measured using a colorimetric urease method, producing a quantifiable color change, while serum creatinine was assessed through the Jaffe reaction, forming a detectable complex with picric acid. The eGFR was calculated using the Modification of Diet in Renal Disease (MDRD) equation, a standard method for evaluating renal impairment, accounting for age, sex, and race [50].

The mean and 90th-percentile values of changes in eGFR were used as the primary criteria for evaluating the safety of herbal medicine on kidney function. Furthermore, we also evaluated cases where eGFR was less than 60 and cases where eGFR decreased by 20% or more, as these are indicative of possible renal function deterioration. A comprehensive analysis of both subjective complaints and laboratory findings reported in the RCTs was conducted to ensure a thorough evaluation of the safety of the herbal medicines.

The protocols of the 6 RCTs analyzed in this study were approved by the Institutional Review Boards and were conducted in accordance with the Declaration of Helsinki. Specifically, the protocol approval numbers for each of the RCTs are as follows: DJMC 2009-01 and DC13MDMT0041 for CGX, DJOMC-136-01 and DJDSKH-17-DR-25–2 for BST, DJDSKH-17-DR-03 and DIRB-00139-3 for Myelophil, and DJOMC-33-1 and DJOMC-51 for *Panax ginseng* extract. All participants provided their informed consent for the publication of research utilizing the pertinent data.

### 4.2. Preparation of Herbal Prescription and Placebos

In an analysis of data from 6 RCTs focusing on kidney function, we estimated the risk of kidney injury associated with four distinct herbal medicines. Myelophil [24], BST [25,26], and CGX [27] demonstrated potential benefits for symptoms of CFS, functional dyspepsia, and liver fibrosis, respectively. Myelophil is used for CFS, BST for functional dyspepsia, and CGX targets liver fibrosis. Additionally, *Panax ginseng* extract was tested for its ability to alleviate mental fatigue and oxidative stress markers in healthy volunteers and patients with chronic fatigue, showing promising results [28,29].

Specifically, the compositions and dosages of these herbal medicines are as follows: Myelophil capsules are administered in daily doses of 2 g for 12 weeks. Each capsule contains 1.389 g of both *Astragalus membranaceus* and *Salvia miltiorrhiza*, formulated as 30% ethanol extracts. BST syrup, administered as a 10 g daily dose for four weeks, includes extracts of *Pinellia ternata* (1.178 g), *Scutellaria baicalensis* (0.840 g), *Panax ginseng* (0.803 g), *Glycyrrhiza uralensis* (0.732 g), *Ziziphus jujuba* (0.512 g), *Zingiber officinale* (dried, 0.500 g), *Coptis chinensis* (0.133 g), and *Zingiber officinale* (fresh, 0.077 g), all derived through boiling-water extraction. CGX tablets, taken twice daily at 1 g or 2 g doses for 12 weeks, are composed of *Artemisia capillaris* (0.660 g), *Trionyx sinensis* (0.660 g), *Raphanus sativus* (0.660 g), *Atractylodes macrocephala* (0.400 g), *Poria cocos* (0.400 g), *Alisma orientalis* (0.400 g), *Atractylodes chinensis* (0.400 g), *Salvia miltiorrhiza* (0.400 g), *Polyporus umbellatus* (0.260 g), *Poncirus trifoliata* (0.260 g), *Amomum villosum* (0.260 g), *Glycyrrhiza uralensis* (0.130 g), and *Aucklandia lappa* (0.130 g). These ingredients are all extracted using boiling water. For *Panax ginseng* extract, there are two types of capsules: one contains 1 g and the other 2 g of a 20% ethanol extract from 4-year-old *Panax ginseng* roots. Participants are administered a daily dosage corresponding to their assigned group, 1 g for the low-dose group or 2 g for the high-dose group, over a period of 4 weeks. 

Each herbal formulation was manufactured in accordance with Korean Good Manufacturing Practices (KGMPs), and the presence of major compounds was verified using molecular fingerprinting techniques, including Ultra-High-Performance Liquid Chromatography (UHPLC) analysis. Placebos for each medication were meticulously designed to replicate the appearance and taste of their respective medicines, employing blends of starch, lactose, flavorings, and other inert components to ensure study integrity. Detailed information on the composition is provided in Supplementary Table S1, while molecular fingerprinting details for the major compounds of each medication are shown in Supplementary Tables S2 and S3.

### 4.3. Participants Criteria

In the 6 RCTs analyzed, participant selection was tailored to the specific condition under investigation, ensuring the accuracy and relevance of the findings. For the CGX trial, we included adults with chronic liver disorders, indicated by a Liver Stiffness Measurement (LSM) score between 5.5 and 16 kPa (a non-invasive measure of liver fibrosis), excluding patients at high risk for liver cirrhosis (LSM > 16 kPa) or with severe hepatic complications. Myelophil targeted adults diagnosed with CFS but excluded those requiring ongoing medication for other conditions or with recent fatigue-causing diseases. BST was directed at adults with dyspepsia symptoms, as defined by the ROME III criteria, but excluded individuals with major gastrointestinal surgeries or severe systemic disorders. The ginseng trials included healthy individuals and those suffering from chronic fatigue, explicitly excluding participants with significant health anomalies, lifestyle risks, or severe psychological conditions. The detailed inclusion and exclusion criteria applied in the 6 RCTs are presented in Supplementary Tables S4–S8. 

### 4.4. Statistical Analysis 

The independent-sample *t*-test was utilized to compare changes in renal function-related values (BUN, serum creatinine, and eGFR) between the intervention and placebo groups. Categorical variables were analyzed using the chi-square test. This study employed a Per Protocol (PP) analysis to examine changes in renal function, while an Intention-to-Treat (ITT) analysis was used to assess adverse events. Statistical significance was determined at a *p*-value threshold of less than 0.05. All statistical analyses were conducted using GraphPad Prism 10 software (Dotmatics, Boston, MA, USA).

## 5. Conclusions

In conclusion, our current data provide initial evidence that professional prescriptions of herbal medicines exhibit a favorable safety profile for renal function. This offers a valuable resource for addressing safety concerns related to the use of herbal remedies, with a particular focus on kidney function. However, further research involving a larger sample size and more rigorous detection criteria is necessary to conclusively establish the safety of herbal medicines for kidney function and accurately determine the incidence of HIKI.

## Figures and Tables

**Table 1:** Characteristics of participants.

	Intervention Group	Placebo Group	Total
	Male/Female	Male/Female	Male/Female
Number participants (%)	240 (59.0%) 89/151	167 (41.0%) 53/114	407 142/265
Mean age (year)	44.0 ± 10.7 46.0 ± 11.9/42.9 ± 9.7	44.5 ± 11.8 46.7 ± 11.5/43.5 ± 11.9	44.2 ± 11.1 46.3 ± 11.7/43.1 ± 10.7
Mean BMI	22.7 ± 3.0 23.6 ± 2.8/22.2 ± 2.9	23.1 ± 2.9 23.9 ± 3.0/22.7 ± 2.9	22.9 ± 3.0 23.7 ± 2.9/22.4 ± 2.9
Intervention (weeks of treatment, daily dose gram/adult) and participants (male/female)			
CGX (12, 1 or 2)	42 (34/8)	23 (18/5)	65 (52/13)
Myelophil (12, 2)	48 (16/32)	49 (10/39)	97 (26/71)
BST (4, 10)	40 (12/28)	39 (12/27)	79 (24/55)
Ginseng (4, 1 or 2)	110 (27/83)	56 (13/43)	166 (40/126)

Note: Data for continuous variables are shown as mean ± standard deviation, and for categorical variables, data are shown as numbers.

**Table 2:** Changes of renal function values.

Participants (N.) (Int. vs. Plac.)	Before (Top) and after Treatment (Bottom)					
	BUN (mg/dL)		Creatinine (mg/dL)		eGFR (mL/min)	
	Intervention	Placebo	Intervention	Placebo	Intervention	Placebo
Total	13.1 ± 3.3	13.5 ± 3.5	0.81 ± 0.17	0.81 ± 0.17	107.2 ± 26.7	105.8 ± 25.5
(240 vs. 167)	13.5 ± 7.0	13.3 ± 3.4	0.83 ± 0.18	0.84 ± 0.17	105.2 ± 28.5	102.8 ± 26.7
Male	13.9 ± 3.5	14.3 ± 3.2	0.91 ± 0.16	0.92 ± 0.18	93.6 ± 19.7	93.2 ± 20.5
(89 vs. 53)	13.7 ± 4.2	13.4 ± 3.0	0.91 ± 0.19	0.94 ± 0.18	95.9 ± 24.4	91.8 ± 23.5
Female	12.6 ± 3.0	13.1 ± 3.6	0.75 ± 0.14	0.76 ± 0.14	115.4 ± 27.1	111.6 ± 25.5
(151 vs. 114)	12.6 ± 3.4	13.2 ± 3.6	0.78 ± 0.16	0.79 ± 0.15	110.8 ± 29.4	108.0 ± 26.7
CGX	14.4 ± 3.5	15.1 ± 2.5	0.90 ± 0.18	0.89 ± 0.20	92.1 ± 21.7	95.0 ± 25.2
(44 vs. 23)	15.1 ± 4.3	14.6 ± 2.3	0.89 ± 0.15	0.90 ± 0.15	93.6 ± 21.2	91.6 ± 17.2
BST	14.0 ± 4.5	13.4 ± 3.2	0.81 ± 0.22	0.82 ± 0.19	97.2 ± 31.7	92.5 ± 23.1
(40 vs. 39)	13.8 ± 4.8	13.9 ± 3.6	0.81 ± 0.23	0.86 ± 0.16	96.2 ± 28.3	86.1 ± 20.2
Myelophil	12.3 ± 2.4	13.0 ± 3.9	0.77 ± 0.15	0.77 ± 0.13	117.3 ± 28.6	116.8 ± 24.1
(48 vs. 49)	12.5 ± 3.4	13.0 ± 3.4	0.76 ± 0.16	0.80 ± 0.16	121.8 ± 33.3	112.9 ± 24.0
Ginseng	12.6 ± 2.7	13.3 ± 3.5	0.80 ± 0.14	0.82 ± 0.16	112.3 ± 9.2	110.7 ± 23.2
(110 vs. 56)	13.1 ± 9.2	12.6 ± 3.6	0.85 ± 0.16	0.83 ± 0.20	105.6 ± 25.2	110.3 ± 29.3

Note: Values represent mean ± standard deviation. The table compares the kidney function-related values before and after treatment with herbal medicines in both the intervention and placebo groups. No statistically significant differences were observed in the changes in BUN, serum creatinine, and eGFR between the intervention and placebo groups after treatment.

**Table 3:** Detailed analysis of eGFR change.

Sub-Group	Intervention Group (*n* = 240)	Placebo Group (*n* = 167)	Statistics (*p* Value)
Participants in 90th-percentile Analysis (male/female)	22 (2/20)	18 (2/16)	0.591
eGFR in 90th-percentile analysis (mL/min)			
- Before treatment - After treatment - Changed value	141.0 ± 17.9 99.1 ± 13.1 41.9 ± 12.7	140.4 ± 21.7 99.1 ± 21.8 41.3 ± 7.7	0.835
Participants with eGFR < 60			
- Before treatment - After treatment	4 1	3 4	0.939 0.075
Participants with ≥20% reduction in eGFR			
- Total - With eGFR < 60 after treatment	44 (18.3%) 0	35 (21.0%) 2	0.510 0.185
eGFR in ≥20% reduction group (mL/min)			
- Before treatment - After treatment - Changed value	123.2 ± 24.5 90.5 ± 15.1 32.7 ± 13.4	123.0 ± 27.1 89.3 ± 22.0 33.6 ± 10.0	0.736

Note: Participants in the 90th-percentile analysis are those who experienced the greatest changes in eGFR. Statistical analysis was performed using chi-square tests for categorical data and independent-sample *t*-tests for continuous data.

## Data Availability

Data will be made available on request.

## Supplementary Materials

The following supporting information can be downloaded at: https://www.mdpi.com/article/10.3390/ph17050544/s1, Table S1. Components of Herbal prescriptions. Table S2. Molecular Fingerprinting of Myelophil and CGX. Supplementary Table S3. Molecular Fingerprinting of BST and Ginseng Extract. Table S4. Inclusion & Exclusion Criteria for CGX RCT [27]. Table S5. Inclusion & Exclusion Criteria for Myelophil RCT [24]. Table S6. Inclusion & Exclusion Criteria for BST RCTs [25,26]. Table S7. Inclusion & Exclusion Criteria for Ginseng RCT for Healthy Participants [28]. Table S8. Inclusion & Exclusion Criteria for Ginseng RCT for Chronic Fatigue [29].
