# Supplementary material for: Evaluating the Safety of Herbal Medicine on Renal Function: A Comprehensive Analysis from Six Randomized Controlled Trials Conducted with Four Formulations from Traditional Korean Medicine
Source: Pharmaceuticals (Basel). 2024 Apr 23;17(5):544. doi: 10.3390/ph17050544 (PMC11124499; doi:10.3390/ph17050544)
Supplement: Supplementary file 1 [file pharmaceuticals-17-00544-s001.zip › pharmaceuticals-2971900-supplementary.pdf]

Supplementary Table S1. Components of Herbal prescriptions

**CGX**		**BST**	
Scientific name	Amount (g)	Scientific name	Amounts (g)
Artemisia capillaries Thunberg	0.660	*Pinellia ternata* (Thunb.) Makino	1.178
Trionyx sinensis Wiegmann	0.660	*Scutellaria baicalensis* Georgi	0.840
Raphanus sativus Linne	0.660	*Panax ginseng* C.A. Meyer	0.803
Atractylodes macrocephala Koidz	0.400	*Glycyrrhiza uralensis* Fisch	0.732
Poria cocos Wolf	0.400	*Ziziphus jujuba* Mill	0.512
Alisma orientalis (*Sam.*) Juzepczuk	0.400	*Zingiber officinale* Roscoe (dried)	0.500
Atractylodes chinensis Koidzumi	0.400	*Coptis chinensis* Franch	0.133
Salvia miltiorrhiza Bunge	0.400	*Zingiber officinale* Roscoe (fresh)	0.077
Polyporus umbellatus Fries	0.260	**Myelophil**	
Poncirus trifoliate Rafin	0.260	Scientific name	Amounts (g)
Amomum villosum Lour	0.260	*Astragalus membranaceus* Fisch	1.389
Glycyrrhiza uralensis Fisch	0.130	*Salvia miltiorrhiza* Bunge	1.389
Aucklandia lappa Decne	0.130	**Ginseng Extract Capsule**	*Panax ginseng* C.A. Meyer 1 or 2g

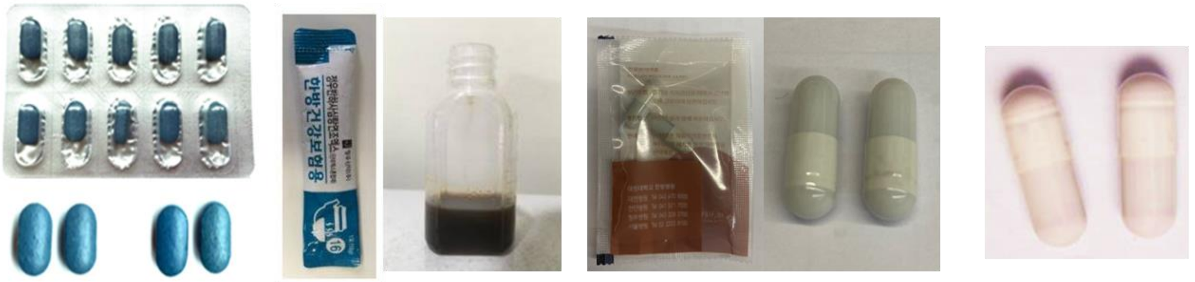

CGX tablet | BST syrup | Myelophil capsule | Ginseng capsule

Supplementary Table S2. Molecular Fingerprinting of Myelophil and CGX

Myelophil: (A) Myelophil and five standards were subjected to UHPLC analysis.
(B) Myelophil and four major compounds were quantitative analyzed by LC/MS.

A

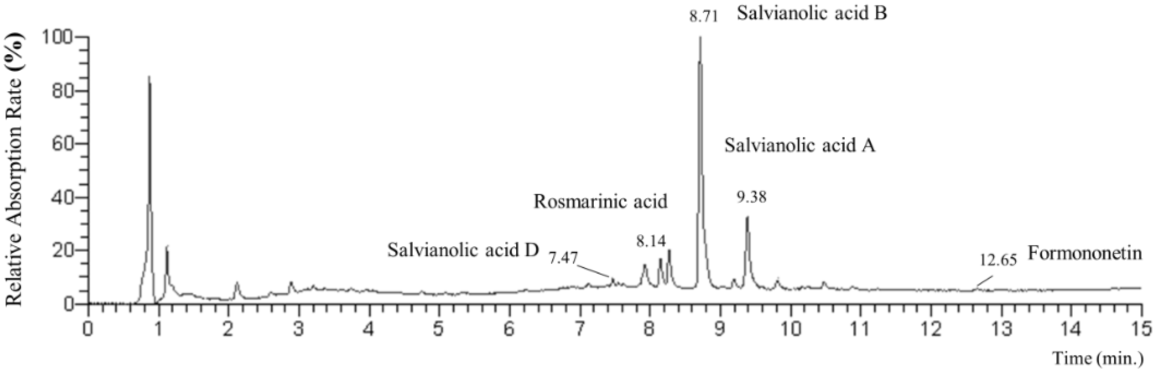

B

Compounds	Quantitative Analysis (mg/g of Myelophil)		
	Astragali Radix	Salviae Radix	Myelophil
Astragaloside IV	1.81 ± 0.01	Not detected	0.87 ± 0.01
Formononetin	0.77 ± 0.01	Not detected	0.21 ± 0.01
Salvianolic acid B	Not detected	86.89 ± 0.19	36.21 ± 0.04
Rosmarinic acid	Not detected	2.53 ± 0.01	1.03 ± 0.01

For detailed information on molecular fingerprinting analysis of Myelophil, please refer to Reference [24].

CGX: (A) CGX and its nine of main compounds were subjected to HPLC.
(B) The contents of each reference compounds in CGX.

A

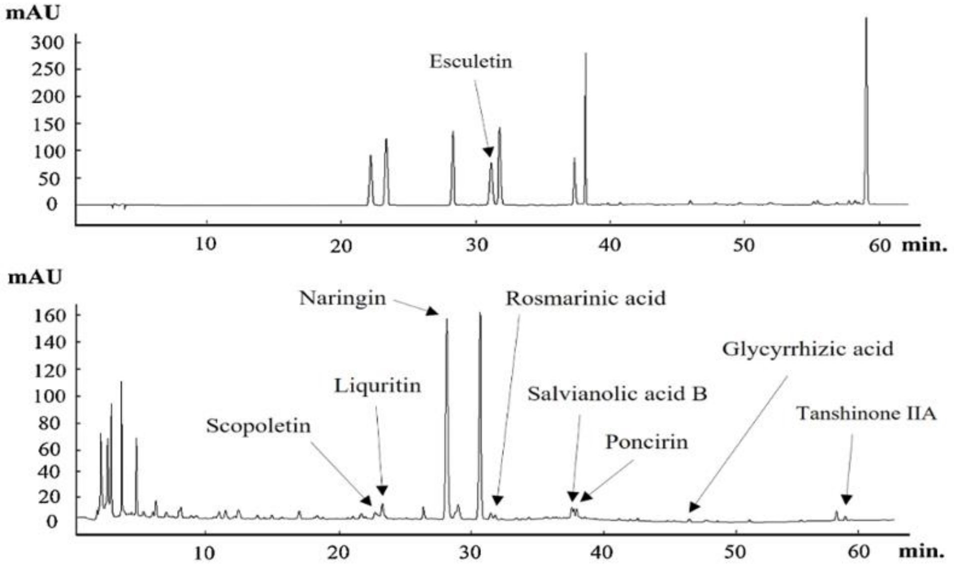

B

	Scopletin	Liquritin	Naringin	Esculetin	Rosmarinic acid	Salvianolic acid B	Poncirin	Glycyrrhiz ic acid	Tanshinone IIA
Retention time (min)	22.13	23.26	28.30	31.21	32.05	37.63	38.22	46.38	59.12
Mean (μg/g of CGX)	5.064	17.927	228.792	N.D.	7.153	21.864	6.632	51.452	1.823
SD	0.136	0.118	6.496	N.D.	0.077	0.313	0.104	0.858	0.130

For detailed information on molecular fingerprinting analysis of CGX, please refer to Reference [27].

Supplementary Table S3. Molecular Fingerprinting of BST and Ginseng Extract

BST: Fingerprint analysis of BST using ultra-high-performance liquid chromatography-tandem mass spectrometry (UHPLC-MS/MS)

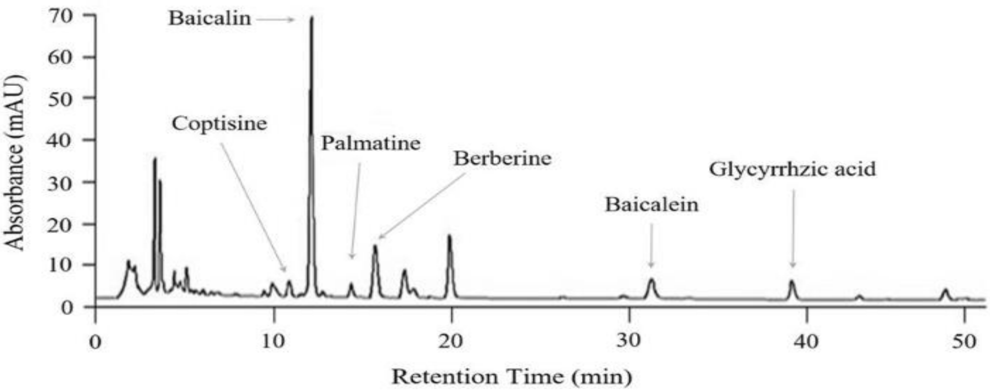

For detailed information on molecular fingerprinting analysis of BST, please refer to Reference [25].

Ginseng Extract: Compositional analysis of *Panax Ginseng* by HPLC.

Protopanaxadiol	mg/g	Protopanaxatriol	mg/g
Rb3	6.33	Rg1	7.22
Rb1	5.14	Re	2.21
Rb2	3.60	Rg2	0.67
Rc	2.61	Rh1	0.58
Rg3	1.08	-	
Rd	0.43	Others	825.8
Rh2	0.002	Crude saponins	144.9

Panax Ginseng (1g) was dissolved in 90% methanol and subjected to HPLC analysis. For detailed methodology, refer to Reference [29].

Supplementary Table S4. Inclusion & Exclusion Criteria for CGX RCT [27]

The inclusion criteria are as follows
1) Participants who are 18 - 65 years old
2) Participants with chronic liver disorder caused by alcohol abuse or viral infection (Hepatitis B or Hepatitis C)
3) Participants who underwent liver fibrosis with 5.5 kPa to 16 kPa of Liver Stiffness Measurement (LSM) value using Fibroscan
4) Participants who are mentally healthy to follow the protocol properly
5) Participants who can give informed consent to participate in this trial
The exclusion criteria are as follows
1) Participants who take immunosuppressive drugs, cytotoxic or hormone therapy
2) Participants with a history of ascites, variceal bleeding, or hepatic encephalopathy
3) Participants with marked liver cirrhosis on ultrasonography or CT scan
4) Participants diagnosed with esophageal varices on endoscopy
5) Participants with jaundice (Total bilirubin > 3mg/dl) or bleeding tendency (INR > 2.0)
6) Participants whose AST or ALT is more than 5 times of ULN
7) Participants with low hemoglobin (< 10g/dl), low number of platelet (< 80,000/mm3), or high serum creatinine (> 1.2-fold of ULN)
8) Participants who use currently alcohol or drugs
9) Participants who have severe disorders of heart, lung kidney, blood, gall-bladder, or allergy
10) Participants with a history of cancer
11) Woman who are in period of pregnancy or lactation, or planning to have a baby
12) Participants whose BMI is more than 30
13) Participants who cannot understand and cannot follow this trial
14) Participants who take ursodesoxycholic acid, silymarin, biphenyl dimethyl dicarboxylate, malotilate, colchicine, prophylthiouracil (PTU), or anti-inflammatory agent

ULN: upper limit of normal, BMI: body mass index

Supplementary Table S5. Inclusion & Exclusion Criteria for Myelophil RCT [24]

The inclusion criteria are as follows
1) Participants who are 18 - 65 years old<br>2) Participants who meet the definition of CFS according to CDC Criteria<br>Referred for severe chronic fatigue lasting more than 6 months, unrelieved by rest, and not attributed to other medical conditions. Additionally, the presence of at least four of the specified symptoms is required<br>① Postexertional malaise lasting more than 24 hours<br>② Unrefreshing sleep<br>③ Difficulty with thinking and memory<br>④ Muscle pain<br>⑤ Multiple joint pain without joint swelling or redness<br>⑥ Headache of a new type, pattern, or severity<br>⑦ Cervical or axillary lymphadenopathy<br>⑧ Sore throat
The exclusion criteria are as follows
1) Participants who take medication for chronic illness.<br>2) Participants who have diseases that induce chronic fatigue within the past 6 months.<br>For example, hypothyroidism, anemia, or psychiatric disorders, etc.<br>3) Participants whose hemoglobin is lower than 12g/dL in men, 11g/dL in women.<br>4) Participants whose AST or ATL is more than 2 times the upper limit of normal,<br>or creatinine is more than 1.2 times the upper limit of normal.<br>5) Participants with high-grade fatty liver on ultrasonography.<br>6) Participants with a history of cancer.<br>7) Participants who work at night.<br>8) Participants who drink more than twice a week.<br>9) Participants whose BMI is less than 16.5 or more than 30.<br>10) Participants who have undergone organ transplantation or take immunosuppressive medication.<br>11) Participants who have been treated for chronic fatigue during the last 2 months.<br>For example, medication, cognitive behavioral or exercise therapy.<br>12) Women who are pregnant or lactating.<br>13) Participants whose BDI is more than 25<br>14) Participants whose STAI is more than 60.<br>15) Participants who cannot understand and cannot follow this trial.

CFS: Chronic Fatigue Syndrome, CDC: US Centers for Disease Control and Prevention, BMI: Body Mass Index, BDI: Beck Depression Inventory), STAI: State-Trait Anxiety Inventory

Supplementary Table S6. Inclusion & Exclusion Criteria for BST RCTs [25,26]

The inclusion criteria are as follows
1) Participants who are 19 - 75 years old
2) Experiencing dyspepsia symptoms, qualifying under the ROME III criteria
3) Symptoms include early satiety, postprandial fullness, and epigastric pain or burning
4) Symptoms not due to structural disease as confirmed by medical assessment, including upper endoscopy
5) Duration of symptoms for more than 3 months with onset at least 6 months before enrollment Presence of two or more moderate symptoms: epigastric pain, discomfort, stuffiness, fullness, burning, postprandial fullness, early satiety, and nausea
6) A total symptom score of at least 6 points across eight symptoms, scored as: 0 (mild), 1 (moderate), 2 and 3 (severe)
7) Excess pattern determined using an instrument of pattern identification for FD (applied only in one of the two BST RCTs [25])
8) Participants who are mentally healthy to follow the protocol properly
9) Participants who can give informed consent to participate in this trial
**The exclusion criteria are as follows**
1) Participants with History of gastrointestinal surgery, except for appendectomy
2) Participants who have Gastrointestinal bleeding, intestinal obstruction, gastrointestinal perforation, colorectal cancer, duodenitis, or stomach cancer.
3) Participants with Severe hepatic dysfunction, congestive heart failure, renal failure
4) Participants whose AST or ATL is more than 2 times the upper limit of normal,<br>or creatinine is more than 1.2 times the upper limit of normal.
5) Participants who have used NSAIDs, corticosteroids, or investigational study drugs within 30 days before study entry
12) Woman who are pregnant or lactating.
15) Participants who cannot understand and cannot follow this trial.

NSAIDs: nonsteroidal anti-inflammatory drug

Supple. Table S7. Inclusion & Exclusion Criteria for Ginseng RCT for Healthy Participants [28]

The inclusion criteria are as follows
1) Participants aged between 20 and 60 years, self-identified as healthy.<br>2) Participants who are mentally helthy to follow the protocol properly.<br>3) Participants who can give informed consent to participate in this trial
The exclusion criteria are as follows
1) Participants whose BMI is more than 30<br>2) Participants with low hemoglobin (less than 10g/dl), a low platelet count (less than 80,000/mm^3), or elevated serum creatinine (more than 1.2 times the upper limit of normal).<br>3) Current users of alcohol or drugs.<br>4) Participants whose AST or ALT levels are more than twice the upper limit of normal, or whose creatinine levels exceed 1.2 times the upper limit of normal.<br>5) Women who are pregnant or lactating, including those trying to conceive during the study period.<br>6) Participants currently taking any form of supplements or health functional foods, including but not limited to vitamins, minerals, and herbal supplements, to avoid potential interaction effects.<br>7) Participants who cannot understand and cannot follow this trial.

ULN: upper limit of normal, BMI: body mass index

Supplementary Table S8. Inclusion & Exclusion Criteria for Ginseng RCT for Chronic Fatigue [29]

The inclusion criteria are as follows
1) Participants aged 20-65 years experiencing chronic fatigue for more than 6 months<br>2) Participants diagnosed with Idiopathic Chronic Fatigue (ICF), characterized by experiencing three or fewer of the eight symptoms typical of Chronic Fatigue Syndrome (CFS) without any other medical explanation.<br>3) Participants who can give informed consent to participate in this trial
**The exclusion criteria are as follows**
1) Participants with any hematological or radiological abnormalities related to fatigue<br>2) Participants who have diseases that induce chronic fatigue within the past 6 months.<br>For example, hypothyroidism, anemia, or psychiatric disorders, etc.<br>3) Participants whose hemoglobin is lower than 12g/dL in men, 11g/dL in women.<br>4) Participants whose AST or ATL is more than 2 times the upper limit of normal,<br>or creatinine is more than 1.2 times the upper limit of normal.<br>5) Participants with high-grade fatty liver on ultrasonography.<br>6) Participants with a history of cancer.<br>7) Participants who work at night.<br>8) Participants who drink more than twice a week.<br>9) Participants whose BMI is less than 16.5 or more than 30.<br>10) Participants who have undergone organ transplantation or take immunosuppressive medication.<br>11) Participants who have been treated for chronic fatigue during the last 2 months.<br>For example, medication, cognitive behavioral or exercise therapy.<br>12) Woman who are pregnant or lactating. Woman who have tried to get pregnant<br>during childbearing age.<br>13) Participants whose BDI is more than 25<br>14) Participants whose STAI is more than 60.<br>15) Participants who cannot understand and cannot follow this trial.

BMI: Body Mass Index, BDI: Beck Depression Inventory, STAI: State-Trait Anxiety Inventory
